# GPR15L is an epithelial inflammation-derived pruritogen

**DOI:** 10.1126/sciadv.abm7342

**Published:** 2022-06-15

**Authors:** Pang-Yen Tseng, Mark A. Hoon

**Affiliations:** Molecular Genetics Section, National Institute of Dental and Craniofacial Research/NIH, 35 Convent Drive, Bethesda, MD 20892, USA.

## Abstract

Itch is an unpleasant sensation that often accompanies chronic dermatological conditions. Although many of the itch receptors and the neural pathways underlying this sensation are known, the identity of endogenous ligands is still not fully appreciated. Using an unbiased bioinformatic approach, we identified GPR15L as a candidate pruritogen whose expression is robustly up-regulated in psoriasis and atopic dermatitis. Although GPR15L was previously shown to be a cognate ligand of the receptor GPR15, expressed in dermal T cells, here we show that it also contributes to pruritogenesis by activating Mas-related G protein–coupled receptors (MRGPRs). GPR15L can selectively stimulate mouse dorsal root ganglion neurons that express Mrgpra3 and evokes intense itch responses. GPR15L causes mast cell degranulation through stimulation of MRGPRX2 and Mrgprb2. Genetic disruption of GPR15L expression attenuates scratch responses in a mouse model of psoriasis. Our study reveals unrecognized features of GRP15L, showing that it is a potent itch-inducing agent.

## Teaser

GPR15L is a potent endogenous keratinocyte-released pruritogen that activates Mrgpr-itch receptors.

## INTRODUCTION

Itch is a common and often debilitating symptom for patients with inflammatory skin disorders including atopic dermatitis (AD) and psoriasis (*1*, *2*). Besides itch, lesioned skin often shows localized allergic reactions including redness, swelling, and rashes. Although it has long been known that histamine released from degranulating mast cells (*3*–*6*) can cause itch through activation of a neural itch circuit (*7*), antihistamine treatment often fails to alleviate symptoms in AD or psoriasis patients, suggesting the involvement of the histamine-independent pathway and insinuating the existence of additional endogenous itch-inducing agents (*2*, *8*, *9*). In some types of itch, it was shown that leukotriene and other substances may be such an agent released by mast cells and basophils (*10*, *11*). However, it seems likely that there are additional unidentified substances that are responsible for itch particularly in dermal conditions associated with chronic itch.

Inflammation is an important skin reaction evoked to various challenges. Among these responses, the release of various damage-associated molecular pattern (DAMP) agents causes stimulation in the innate immune system (*12*). Another dermal reaction during inflammation is the release of antimicrobial substances. In the skin, these include the release of peptides such as β-defensins, cathelicidin (LL-37), and eosinophil major basic protein (PRG2). Another peptide, C10orf99 (chromosome 10 open reading frame 99), which encodes a 57–amino acid cationic peptide, was implicated as an antimicrobial (*13*). In addition, other studies reported that C10orf99 displays DAMP activity and is a ligand for sushi domain containing 2 (SUSD2) (*14*) and was suggested to play a role in psoriasis pathogenesis (*15*, *16*). Recently, several groups independently showed that C10orf99, as well as the mouse ortholog 2610528A11rik, is a cognate ligand for an orphan G-protein–coupled receptor (GPR15) and renamed it GPR15L (*17*, *18*). Contradicting the suggestion that GPR15L has a role in dermal inflammation, it was reported that the disruption of GPR15 does not produce skin pathogenesis and instead produces an altered skin microbiome and changes in immune cell composition in the skin (*19*). This finding hints that skin inflammation occurs through GRP15-independent pathways. Together, these studies suggest that GPR15L is a DAMP involved in regulating skin homeostasis, but its contribution to inflammation has been questioned. Notably, previous studies have not investigated whether GPR15L is a pruritogen and whether it might be directly involved in inflammation.

Cationic antimicrobial peptides such as LL-37 and β-defensins can cause mast cell degranulation (*20*, *21*) through Mas-related G protein–coupled receptors (MRGPRs). In particular, MRGPRX2 and the mouse ortholog Mrgprb2 are receptors for many cationic molecules (*22*–*25*). Activation of MRGPRs on mast cells can cause immunoglobulin E–independent allergic reactions (pseudo-allergy) including extravasation, itch, and pain (*22*, *26*). Some human and tick β-defensins can activate not only MRGPRX2/Mrgprb2 but also MRGPRX1/Mrgprc11, which are expressed on sensory neurons (*27*).

Here, we investigated which molecules might be endogenous pruritogens in human skin diseases associated with itch and found that GPR15L is a highly cationic peptide that is overexpressed in two different inflammatory dermal diseases. Specifically, we found that in human skin diseases, GPR15L is expressed by inflammatory keratinocytes, and its release can activate several MRGPRs expressed on sensory neurons and mast cells to induce itch and inflammation. Together, our results show that GPR15L can act as an endogenous pruritogen during inflammation through a transduction cascade independent of GPR15.

## RESULTS

### C10orf99 (GPR15L) encodes a highly cationic peptide that is selectively expressed in pruritic skin diseases

**Fig. 1.**
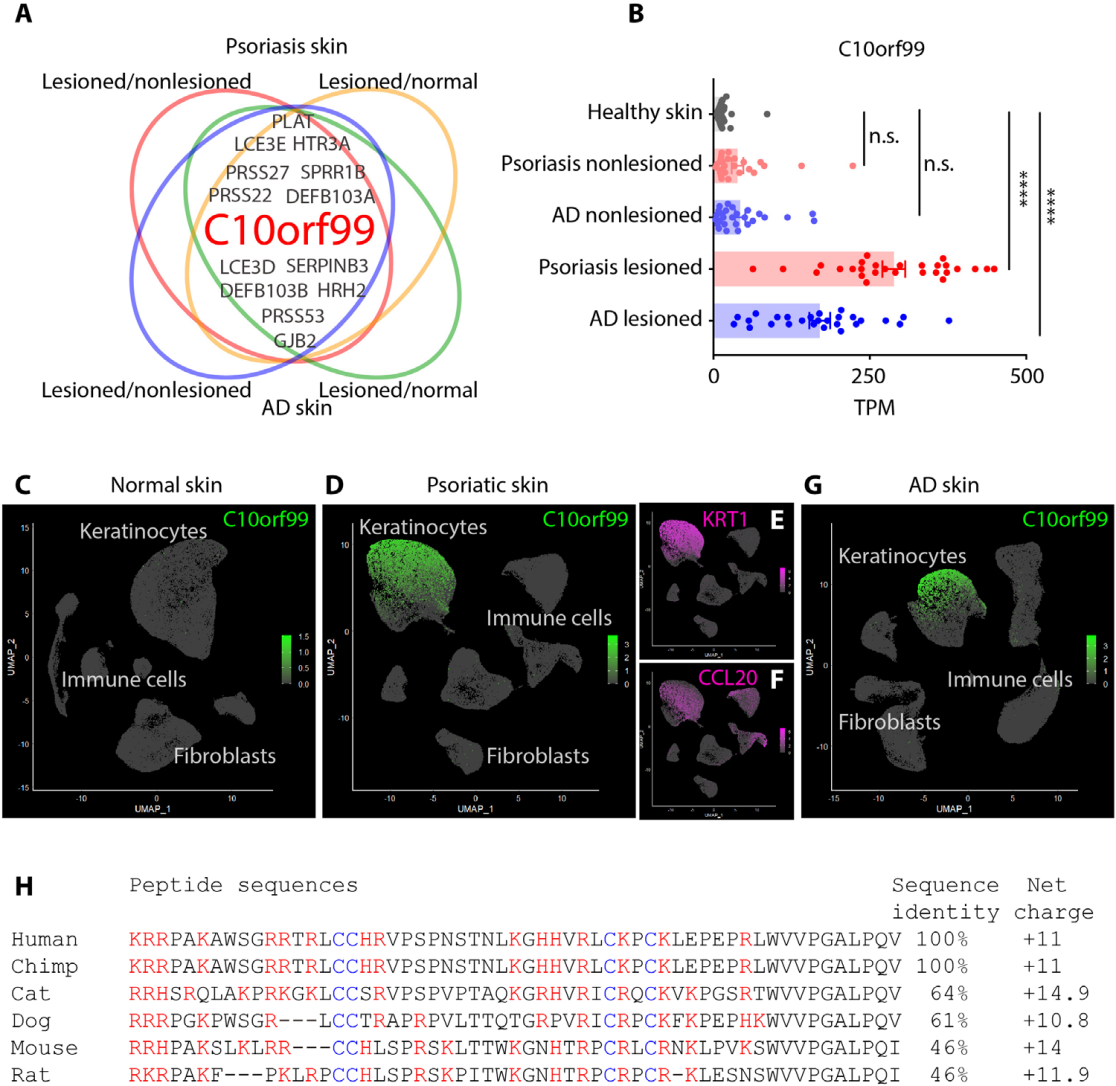
Expression of GPR15L is elevated in inflammatory skin conditions. (**A**) The top 100 up-regulated genes in four differential gene expression analyses of skin samples from AD or psoriasis biopsies (psoriasis/healthy, AD/healthy, psoriasis-lesioned/psoriasis-nonlesioned, and AD-lesioned/AD-nonlesioned) (GSE121212). Venn diagram was generated by jvenn. (**B**) Compared to healthy skin samples (*n* = 38), lesioned skin samples from psoriasis (*n* = 28) and AD (*n* = 27) patients have higher transcript abundances [transcript per million (TPM)] of GPR15L (****P < 0.0001, one-way ANOVA), while the transcript abundances from nonlesioned psoriatic (*n* = 27) or AD (*n* = 27) samples were not statistically higher than healthy samples (*P* = 0.3 and 0.2, respectively, one-way ANOVA); data are presented as means ± SEM. n.s., not significant. (**C** to **G**) Visualization (with the R package Seurat) of GPR15L expression with single-cell RNA-seq analyses from human skin samples. The color bars indicate expression normalized gene level (TPM) at log_10_ scale. (**H**) Comparisons of the sequences of GPR15L from the indicated species showing that these peptides contain high numbers of basic amino acids (red) and conserved cysteines (blue). The net charge values were calculated under the presumption of neutral pH condition.

We wondered whether there are specific agents released by inflamed skin that might be responsible for pruritus in inflammatory skin diseases. To search for candidate genes that could encode potential pruritic mediators, we analyzed publicly available raw RNA-sequencing (RNA-seq) data of skin biopsies collected from psoriasis or AD patients as well as from healthy donors (GSE121212) (*28*). We performed differential gene expression (DGE) analyses of the following pairs of data: psoriasis/healthy, AD/healthy, psoriasis lesioned/nonlesioned, and AD lesioned/nonlesioned. To narrow down the search, we examined the top 100 most differentially expressed genes for each comparison and found *C10orf99* as one of the most up-regulated genes in both psoriasis and AD (Fig. 1A). As expected, the expression of *C10orf99* correlated with skin lesion with lesioned samples displaying higher expression in psoriasis (*13*, *15*, *16*). *C10orf99* expression was also elevated in dermal samples from patients with AD (Fig. 1B). To investigate the source of *C10orf99*, we turned to single-cell RNA-seq data from healthy, psoriatic, or AD skin samples (*29*). This analysis showed that *C10orf99* is mainly expressed by inflammatory differentiated keratinocytes, which display higher expression of differentiated keratinocyte markers *KRT1*, and inflammatory mediator *CCL20* (Fig. 1, C to G) (*30*). Alignment of the protein sequence *C10orf99* showed that the sequence identities across species are not highly conserved, with the sequence identity between human and mouse peptide being 46% (Fig. 1H). However, the positions of charged amino acids relative to cysteine residues, which presumably stabilize the protein, are conserved and are the overall net charge of peptides (+10.8 to +14.9). The calculated net charge values are higher than other cationic antimicrobial peptides including LL-37 (+6), β-defensin 2 (+5.7), and β-defensin 3 (+10.6).

Although the gene name *C10orf99/2610528A11Rik* remains in use, reference transcriptomes (Ensembl GRCh38.v104 and 10X reference GRCh38 2020-A) name this peptide *GPR15L/Gpr15l*, and this name is now the most common in the literature. For this reason, in the remainder of this manuscript, we refer to *C10orf99/2610528A11Rik* as *GPR15L/Gpr15l*.

### GPR15L induces itch and skin vascular dilation

**Fig. 2.**
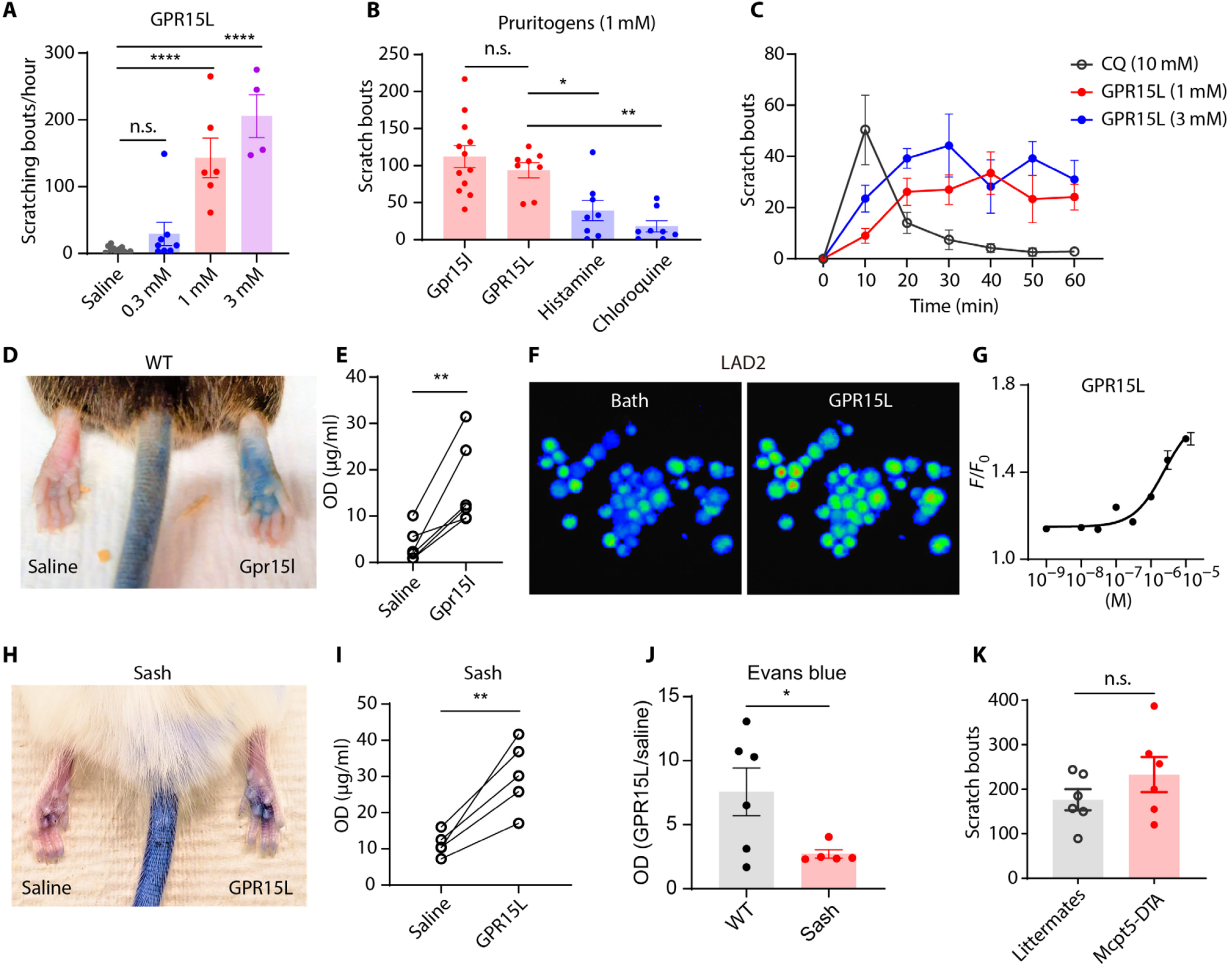
GPR15L is a pruritic, proinflammatory peptide. (**A**) Intradermal delivery of human GPR15L peptide dose-dependently evoked itch when compared to saline injection [*P* = 0.57 in 0.3 mM (*n* = 8), *P* < 0.0001 in 1 mM (*n* = 6) and 3 mM (*n* = 4), one-way ANOVA]. (**B**) Both mouse Gpr15l (1 mM, *n* = 12) and human GPR15L (1 mM, *n* = 8) elicited intense scratching responses that were not distinguishable (*P* = 0.6) but were significantly stronger than histamine (1 mM, *n* = 8, *P* = 0.02) and chloroquine (1 mM, *n* = 8, *P* = 0.001), one-way ANOVA. (**C**) GPR15L (*n* = 6 in 1 mM, *n* = 4 in 3 mM) evoked long-lasting scratching responses when compared to chloroquine (*n* = 5). (**D** and **E**) Evans blue assay showed that Gpr15l (1 mM) induced localized allergic reactions upon intraplanar injection (*n* = 6, *P* = 0.007, paired *t* test). (**F** and **G**) Calcium imaging of human mast cells and LAD2 cells showed that GPR15L (1 μM) triggered increases in intracellular calcium with an EC_50_ of 1.8 ± 0.8 μM (three replicates). (**H** and **I**) GPR15L induced extravasation in Sash mice (*n* = 5, *P* = 0.006, paired *t* test). (**J**) Normalized OD values (GPR15L/saline) showed that sash mice displayed less severe extravasation compared to wild-type (WT) mice (*P* = 0.04, *t* test). (**K**) Upon Gpr15l (1 mM) injection, mice lacking mast cells (Mcpt5-DTA) displayed itch phenotype that was not different from littermates or wild type (*n* = 6, *P* = 0.2, *t* test). All data are presented as means ± SEM.

Mast cells can be activated by various cationic peptides, such as LL-37 and β-defensins, through activation of MRGPRX2 (*23*, *24*, *31*). These peptides are thought to be potent secretagogues because they are highly cationic. Given the high positive net charge of GPR15L (Fig. 1H), we hypothesized that this peptide may also act as a previously unrecognized pruritogen by activating mast cells. In line with our prediction, intradermal injection of GPR15L peptides dose-dependently evoked robust scratching responses in mice (Fig. 2A). Both human GPR15L and mouse Gpr15l evoked intense itch phenotype, and the pruritic potencies of GPR15L (molar concentrations) were higher than that for both histamine and chloroquine (Fig. 2B). GPR15L exhibited long-lasting pruritogenic effects when compared to chloroquine (Fig. 2C). As expected, intraplanar delivery of GPR15L caused increased vascular permeability resulting in extravasation (Fig. 2, D and E). Calcium imaging studies revealed that administration of GPR15L can induce increases in intracellular calcium in LAD2 (Laboratory of Allergic Diseases 2) human mast cells (Fig. 2F) (*32*) with a median effective concentration (EC_50_) of 1.8 ± 0.8 μM (Fig. 2G). To test whether the pruritogenic and proinflammatory effects of GPR15L were through the activation of mast cells, we tested mast cell–deficient mice with the Sash mutation (c-Kit^w-sh^). Sash mice exhibited reduced extravasation compared to wild-type mice (Fig. 2, H and J). However, although the severity of extravasation was attenuated, Sash mice still displayed Evans blue staining after GPR15L treatment (Fig. 2I). This suggests that GPR15L induces the release of non–mast cell mediators. In addition, the itch phenotype evoked by GPR15L was not reduced by mast cell depletion (Fig. 2F) (*33*). This result is in agreement with previous studies that reported that for some compounds that activate mast cells, mast cells are dispensable for itch (*31*, *34*). Collectively, these results suggest that GPR15L may activate sensory neurons to directly evoke itch and additionally may, through the release of CGRP from sensory neurons, indirectly induce vasodilation.

### GPR15L activates itch-selective sensory neurons through MRGPR proteins

**Fig. 3.**
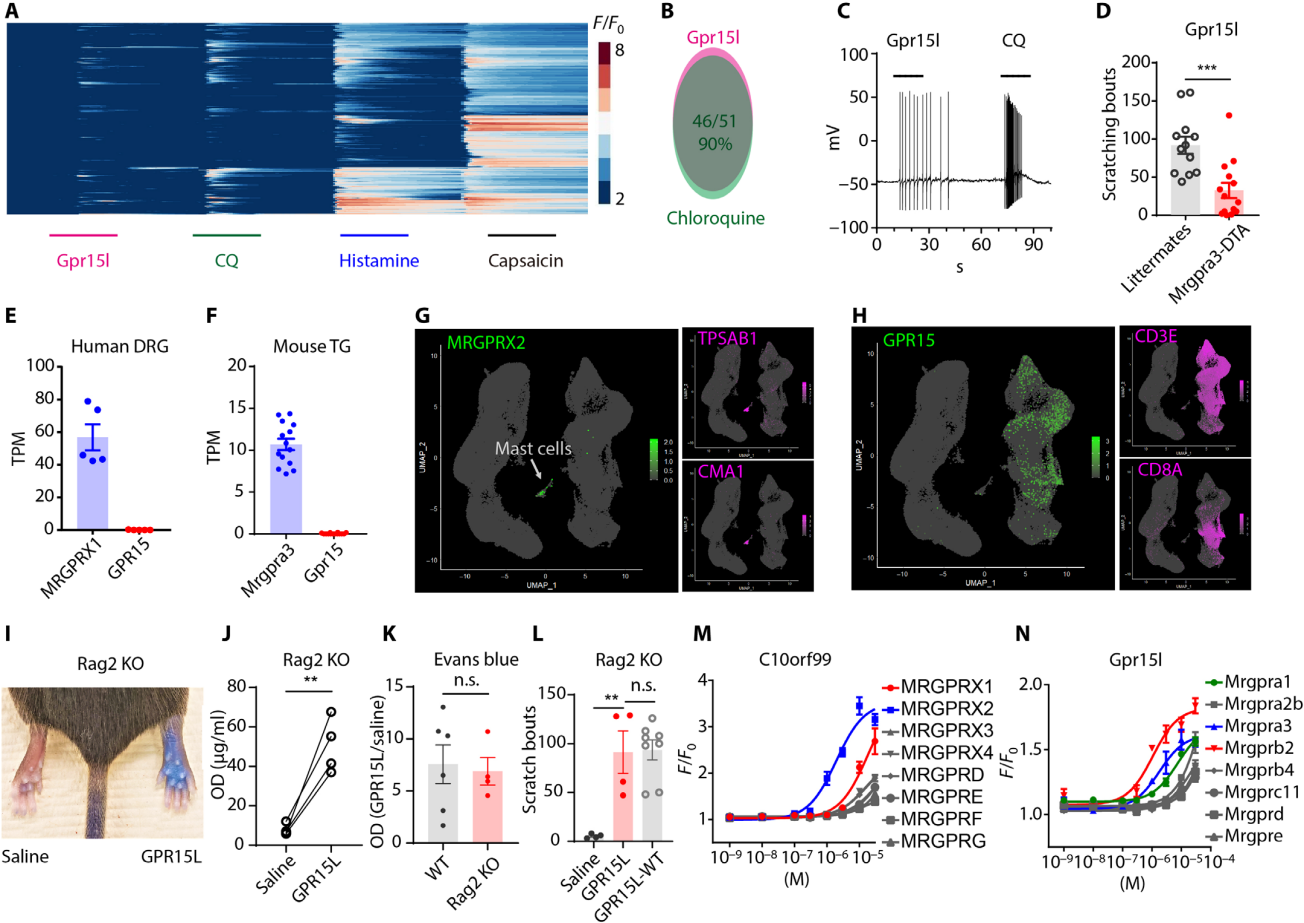
GPR15L activates sensory neurons through MRGPRs. Calcium imaging dissociated DRG neurons expressing GCaMP6f. Neurons (*n* = 245) were sequentially challenged with Gpr15l (1 μM), chloroquine (100 μM), histamine (100 μM), and capsaicin (10 μM) (scale bars, 30 s). (**B**) About 90% of Gpr15l-responsive neurons also responded to chloroquine. (**C**) Whole-cell patch-clamp recording showed that dissociated Mrgpra3-Cre-GFP DRG neurons fired action potentials in response to Gpr15l (1 μM) and chloroquine (100 μM) challenge (*n* = 7/8 neurons). (**D**) Mrgpra3-creDTA (diptheria toxin A) mice exhibit attenuated scratching responses to Gpr15L (1 mM) challenge (*P* = 0.0006, *t* test, *n* = 12). (**E**) Transcript abundances (TPM) of *GPR15* and *MRGPRX1* in human DRG samples (*n* = 5) (*37*). (**F**) Transcript abundance of *Gpr15* and *Mrgpra3* in mouse trigeminal ganglia (*n* = 14, GSE132173) (*38*). (**G** and **H**) Single-cell RNA-seq analysis of human dermal immune cells (CD45^+^, *n* = 84,880 cells) from healthy skin biopsies (*29*) showed that (G) *MRGPRX2* is detected in the mast cell population (indicated with an arrow) that express *tryptase* (*TPSAB1*) and *chymase* (*CMA1*), while *GPR15* is mainly detected in T cells that coexpress CD3E or CD8A (H) but not in mast cells. (**I** and **J**) GPR15L induced extravasation in Rag2 KO mice (*n* = 4, *P* = 0.008, paired *t* test). (**K**) Normalized OD values (GPR15L/saline) showed that the severity of extravasation between Rag2 KO (*n* = 4) and wild-type mice (*n* = 6) was not significantly different (*P* = 0.78, *t* test). (**L**) Intradermal injection of GPR15L evoked intense scratching behavior in Rag2 KO mice (*P* = 0.002, *t* test) that is not statistically different from wild-type mice (*P* = 0.99, *t* test). (**M**) FLIPR screening of HEK293 cells heterologously expressing human MRGPR or (**N**) mouse Mrgpr receptors.

To test whether Gpr15l activates sensory neurons, we performed calcium imaging on dissociated dorsal root ganglion (DRG) neurons expressing the calcium indicator GCaMP6 (DRG neurons transfected with AAV9 (adeno-associated virus serotype 9)-syn-GCaMP6f). Recombinant Gpr15l peptide (1 μM) induced calcium mobility in a subpopulation of neurons. The majority of these neurons also responded to chloroquine, histamine, and capsaicin, suggesting that this peptide activates a specific subtype of itch-selective sensory neurons that express the chloroquine receptor Mas-related G protein–coupled receptor A3 (Mrgpra3) (Fig. 3, A and B) (*35*). Whole-cell patch-clamp recording confirmed that Gpr15l (1 μM) elicited action potentials on genetically labeled Mrgpra3 DRG neurons [Mrgpra3-Cre–enhanced green fluorescent protein (eGFP)] (Fig. 3C, *n* = 7/8 neurons) (*36*). In agreement with Mrgpra3 neurons being activated by Gpr15l, ablation of Mrgpra3 neurons attenuated Gpr15l-induced scratching responses (Fig. 3D). Recently, GPR15L has been designated as the cognate ligand for GPR15 and plays an important role in T cell homeostasis (*17*, *18*); however, whether GPR15 is expressed in DRG is unclear. We examined published bulk RNA-seq data of human DRG (*37*) and mouse trigeminal ganglia (GSE132173) (*38*) and noticed that, compared to MRGPRX1 and Mrgpra3, the expression level of *GPR15*/*Gpr15* in sensory neurons is negligible (Fig. 3, E and F). Because GPR15L can also activate mast cells to induce localized allergic reactions, we wondered whether GPR15 is expressed by mast cells. We reanalyzed single-cell RNA-seq analyses of human dermal immune cells (*29*) and classified these cells into mast cells, lymphoid cells (T cells, natural killer cells, and innate lymphoid cells), and antigen-presenting cells (Langerhans and dendritic cells) (Fig. 3G). This analysis showed that GPR15 is selectively expressed by T cells (CD3^+^ or CD8^+^) (Fig. 3H), while MRGPRX2 is expressed by mast cells (tryptase^+^/chymase^+^) (Fig. 3G). To further test whether GPR15 is required for the itch and proinflammatory effects induced by GPR15L, we tested GPR15L on Rag2 knockout (KO) mice in which mature T and B cells are absent (*39*). In line with the RNA-seq data, GPR15L induced strong localized allergic reactions and scratching responses in Rag2 KO mice, which were indistinguishable from wild-type mice (Fig. 3, I to L). Given that GPR15L can activate mast cells as well as DRG neurons that express MRGPRX2 and Mrgpra3, respectively, we hypothesized that the effects of GPR15L on mast cells and sensory neurons occur through activation of MRGPRs. To test whether GPR15L/Gpr15l can directly activate MRGPRs, we used a fluorescence imaging plate reader (FLIPR) calcium imaging assay to screen human embryonic kidney (HEK) cells heterologously expressing MRGPRs; human MRGPRX1, MRGPRX2, MRGPRX3, MRGPRX4, MRGPRD, MRGPRE, MRGPRF, and MRGPRG (Fig. 3M); and mouse Mrgpra1, Mrgpra2b, Mrgpra3, Mrgprb2, Mrgprb4, Mrgprc11, Mrgprd, and Mrgpre (Fig. 3N). We found that among the eight human MRGPRs, MRGPRX2 displayed the highest potency for GPR15L with an EC_50_ of around 1.9 ± 0.5 μM, which is comparable to the calculated EC_50_ for GPR15L for activation from human mast cells (Fig. 2G). Our results also showed that GPR15L can activate MRGPRX1, with an EC_50_ about 10-fold lower than that for MRGPRX2 (Table 1). In mouse, Mrgpr proteins Mrgprb2 and Mrgpra3 exhibit potencies of 1.1 ± 0.3 and 1.8 ± 0.6 μM, respectively (Table 2), which are comparable to those for human MRGPRX2. Together, these results demonstrate that GPR15L/Gpr15l can activate multiple MRGPRs but are especially potent for MRGPRX2, Mrgprb2, Mrgpra3, and MRGPRX1.

**Table 1.** Human GPR15L potencies on human MRGPRs.

**Human**	**GPR15L**							
**MRGPR**	**X1**	**X2**	**X3**	**X4**	**D**	**E**	**F**	**G**
EC_50_ (μM)	18.1 ± 8	1.9 ± 0.5	71 ± 40	15.7 ± 4	17 ± 3	38 ± 2	19 ± 8	170 ± 193

**Table 2.** Mouse Gpr15l potencies on mouse Mrgprs.

**Mouse**	**Gpr15l**							
**Mrgpr**	**a1**	**a2b**	**a3**	**b2**	**b4**	**c11**	**d**	**e**
EC_50_ (μM)	7.6 ± 1.8	60 ± 45	1.8 ± 0.6	1.1 ± 0.3	24 ± 11	20 ± 12	n.d.	102 ± 97

### GPR15L contributes to inflammatory symptoms and pruritus

**Fig. 4.**
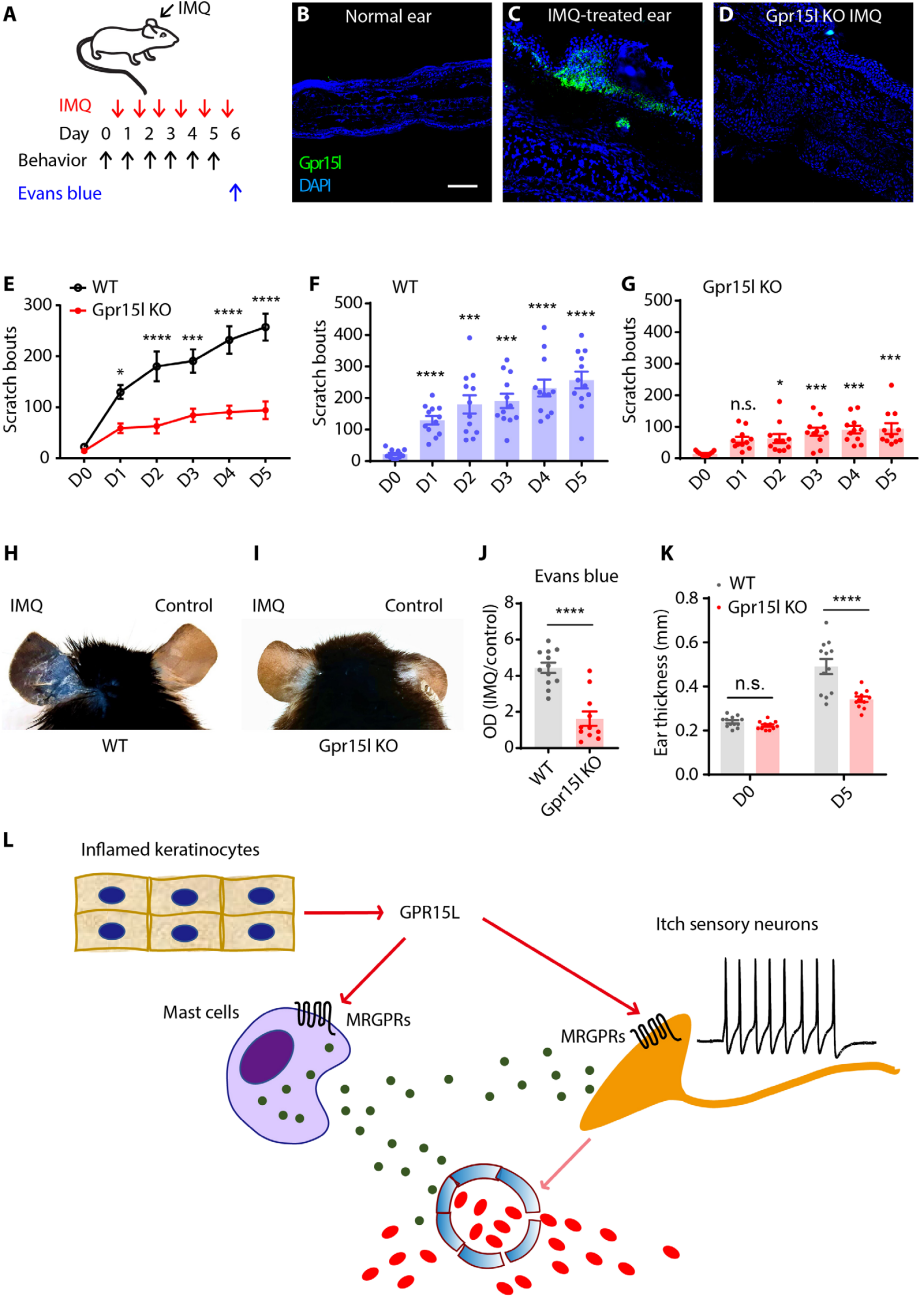
GPR15L contributes to vascular dilation and itch. (**A**) Schematic diagram of experimental design of the mouse model of psoriasis and the timeline for testing behavior and amount of extravasation. Imiquimod (0.05 g) was applied daily to the ear before itch behavioral assay for 5 days. Evans blue (1%, 200 μl) was injected through tail veins at day 6 to evaluate the extent of skin extravasation. (**B** to **D**) In situ hybridization of *Gpr15l* in (B) the untreated ear and (C) the imiquimod-treated ear of wild-type mice for 7 days, and (D) the imiquimod-treated ear (7 days) of Gpr15L null mice. Scale bar, 100 μm. (**E**) Gpr15l KO mice (*n* = 11) exhibited less intense scratching behavior than wild-type animals (*n* = 12) starting from days 1 to 5 (*P* = 0.04, *P* < 0.0001, *P* = 0.0005, *P* < 0.0001, and *P* < 0.0001, respectively, two-way ANOVA). (**F**) Wild-type mice developed intense itch phenotype 1 day after imiquimod treatment and afterward (*n* = 12, *P* < 0.0001, *P* = 0.0006, *P* = 0.0001, *P* < 0.0001, and *P* < 0.0001, respectively, one-way ANOVA). (**G**) Gpr15l KO mice developed less intense itch phenotype (*n* = 11, *P* = 0.053, *P* = 0.030, *P* = 0.0008, *P* = 0.0002, and *P* = 0.0001, respectively, one-way ANOVA). (**H** and **I**) In the Evans blue assay of extravasation, Gpr15l null mice displayed less severe capillary leakage than control mice after 5-day imiquimod treatment (*P* < 0.0001, *t* test). (**K**) Gpr15l KO mice displayed less skin hyperplasia after 5 days of imiquimod treatment (*P* < 0.0001, two-way ANOVA). (**L**) Schematic summary of the mechanisms for GPR15L-mediated itch and vascular leakage.

Given that GPR15L can activate sensory neurons and mast cells and that its expression is greatly increased in inflamed skin, we hypothesized that GPR15L might be an important contributor of inflammatory skin symptoms and pruritus. To test this hypothesis, we assayed mice lacking Gpr15l in an imiquimod-induced psoriasiform dermatitis model. Specifically, we induced a psoriasis-like condition on the ear and examined itch behavioral phenotype directed to the ear and levels of inflammation in the ear skin (Fig. 4A). As expected from our transcriptional analyses, in situ hybridization showed that imiquimod treatment induced robust *Gpr15l* mRNA expression in the hypertrophic epidermis from wild-type mice ears (Fig. 4, B and C), and in Gpr15l null mice, this transcript was not detectable (Fig. 4D). Concordant with our hypothesis for a role of Gpr15l in itch, mice lacking *Gpr15l* exhibited a less intense itch phenotype in this model of psoriasis (Fig. 4, E to G). Furthermore, *Gpr15l* null mice exhibited less severe extravasation (Fig. 4, H to J) and displayed less skin hypertrophy (Fig. 4K) compared to wild-type littermates. Together, these functional in vivo studies establish that GPR15L is a critical keratinocyte-derived peptide that has a major contribution to pruritus and vasodilation (Fig. 4L).

## DISCUSSION

Here, we searched for and investigated the properties of potential endogenous pruritogens in dermal skin conditions associated with itch. We found that the expression of peptide GPR15L is increased in the skin of patients with psoriasis as well as AD (which was not previously reported). Our studies aimed at determining whether GPR15L is a pruritogen that mechanistically and potently stimulates sensory neurons and is a mast cell secretagogue. We showed that these cell activation processes occur via activation of MRGPRs. In addition, our studies demonstrate that GPR15L is an important molecular component in the development of itch in psoriasis.

Previously, GPR15L (C10orf99) was reported to be a candidate gene involved in psoriasis (*15*, *40*), and in translational studies, the expression of GPR15L was used as a therapeutic biomarker (*18*, *41*). In addition, knockdown of Gpr15l (2610528A11Rik) ameliorated imiquimod-induced skin lesions and reduced dermal hypertrophy (*16*). It was also shown that the GPR15L chromosomal locus is one of the most differential methylated regions in psoriatic skin samples (*42*). Our analyses of RNA-seq data from samples from psoriasis patients agree with these previous reports showing that *GPR15L* is one of most up-regulated genes in diseased skin. In addition, our analysis revealed that *GPR15L* expression is elevated in AD lesions compared to controls with the expression level correlated with disease severity (Fig. 1B), suggesting that GPR15L may act in more inflammatory skin diseases than previously recognized. Furthermore, because previous studies on GPR15L focused on its effects on skin hypertrophy, our studies extend knowledge of the function of GPR15L by showing that it produces itch and vasodilation.

A receptor for GPR15L, GPR15, was formerly described (*17*, *18*), but inconsistent with having a role in itch and localized allergic reactions, this receptor is expressed predominantly in T cells. The latter expression pattern was interpreted to suggest that GPR15 is a chemokine receptor and that GPR15L is a chemokine. Because effects on dermal lymphocytes might potentially indirectly affect itch and inflammation by producing barrier dysfunction, some of the effects we observed in our studies may have formally arisen through GPR15 particularly in our studies using the imiquimod model of psoriasis (*19*). However, our demonstration that GPR15L activates MRGPRs, which induces itch, provides an alternate reasonable explanation for the phenotype in GPR15 null mice. In addition, mast cells release multiple chemoattractants that may be involved in the initiation phase of psoriasis and contribute to the reduced itch responses we observed in Gpr15l null mice (*43*). Together, our results strongly suggest that GPR15L has several different receptor and cellular substrates that produce physiologically different reactions.

There is accumulating evidence suggesting that cationic antimicrobial peptides including β-defensins and LL-37 can activate mast cells and sensory neurons through MRGPRs and could be endogenous ligands for MRGPRs (*5*, *23*, *24*, *31*, *44*). The human genome encodes eight *MRGPR* genes, while the mouse genome encodes more than 30 *Mrgpr* genes (*45*–*47*). The functional homology between human and mouse MRGPR proteins is not well defined. Nevertheless, it is acknowledged that there are strong similarities between MRGPRX2 and Mrgprb2 expressed in mast cells (*46*) and between MRGPRX1 and Mrgpra3 expressed by itch-selective sensory neurons (*35*, *36*, *48*). Our screening results in which GPR15L displayed higher potency for MRGPRX2, MRGPRX1, Mrgprb2, and Mrgpra3 correlate with the skin symptoms caused by GPR15L and previous findings that cationic peptides activate these receptors (*22*–*24*, *31*, *44*). GPR15L is not expressed at appreciable levels in normal skin; however, its expression is markedly induced upon inflammation (*16*, *18*, *19*). Under physiological conditions, GPR15L is expressed in many epithelial tissues, including colon, tongue, and cervix (*17*, *18*). Therefore, it was proposed that GPR15L acts as a broad-spectrum antimicrobial peptide against Gram-positive bacteria, fungi, and virus (*13*), providing host defense for the epithelial barrier. Itch is also considered a defensive strategy protecting our skin barrier by removing parasites (*49*, *50*). Given that the sensation of itch arises from perturbations of the skin, GPR15L is likely a pleiotropic multifaceted signaling molecule, which orchestrates responses from the innate immune system (antimicrobial and DAMP), mast cells, and peripheral nerve fibers for host defense.

## MATERIALS AND METHODS

RNA sequence reads were downloaded from Gene Expression Omnibus (https://www.ncbi.nlm.nih.gov/geo/) by the SRA-Toolkit with accession numbers indicated in the text. Sequencing reads were aligned to Ensembl reference transcriptomes GRCh38.v98 (*Homo sapiens*) and GRCm38.v98 (*Mus musculus*) to index files generated by kallisto (*51*). Raw counts and transcript abundances were estimated by kallisto. Data retrieval, reads mapping, and counting were processed on National Institutes of Health (NIH) Biowulf cluster. Raw counts were imported into R and RStudio by tximport (*52*) for differential gene expression analysis by DESeq2 (*53*). Top differentially expressed genes from multiple datasets were plotted and identified using jvenn (*54*). Single-cell RNA-seq data were processed and analyzed with the R package Seurat (*55*–*58*).

### Animals

Experiments using mice followed NIH guidelines and were approved by the National Institute of Dental and Craniofacial Research Animal Care and Use Committee. Eight- to twelve-week-old male and female mice were used: C57BL/6N (Envigo), Gpr15l (2610528A11Rik) KO (#TF3294, Taconic Bioscience Inc.), Sash mutant (c-Kit ^w-sh^) mice (#030764, The Jackson Laboratory), Rag2 KO (recombination activating gene 2 deletion) mice (#033526, The Jackson Laboratory), ROSA-stop-DTA (#009669, The Jackson Laboratory) (*59*), Mrgpra3-Cre-eGFP from X. Dong (*36*), and Mcpt5-Cre (*33*).

### Itch behavior

Behavioral assessment of scratching was conducted as previously described (*60*). Recombinant GPR15L/Gpr15l peptides (Genscript USA Inc. or Biomatek USA LLC) or other pruritogens were intradermally injected (10 μl in saline) into the mouse nape area. Bouts were defined as scratching events directed toward the site of injection from lifting the hind leg from the ground to returning it. Itch responses were quantified by the bouts evoked within 30 min after chemical injection. In some behavioral tests, scratching bouts were automatically counted with the MicroAct system (Neuroscience, Inc.), and the monitor time was extended to 60 min as previously described (*61*). Briefly, a magnet (1 mm in diameter and 3 mm in length) was implanted under the skin of the dorsal hind paw at least 7 days before the behavioral tests (*62*). About 10 min before the tests, mice were placed into a special observation chamber with a ring coil. Vertical movements of the implanted magnets will induce currents, which were recorded. Scratching bouts were determined using MicroAct software (*63*, *64*).

### Extravasation

Evans blue was used to assay extravasation as previously described (*44*). Briefly, Evans blue powder (Sigma-Aldrich) was dissolved in saline to 1% working solution. Approximately 200 to 250 μl (100 mg/kg) of the solution was injected through mouse tail veins. Hind paws were injected subcutaneously with 10 μl of GPR15L/Gpr15l peptide (GenScript USA Inc. and Biomatek USA LLC), and an equal volume of saline was injected into the contralateral paw. Tissues were dissected, air-dried overnight, weighed, and then immersed into 400 μl of formamide overnight. Optical density (OD) values (mg/ml) were measured with a spectrophotometer at 610-nm wavelength. Plasma extravasation values were calculated by normalized OD values with tissue weights.

### Mast cell culture and imaging

LAD2 cells were maintained in StemPro-34 (Gibco) supplemented with recombinant human stem cell factor (100 ng/ml) and penicillin/streptomycin, as described previously (*32*). LAD2 cells were loaded with Fluo-8 AM for 30 min at 37°C, washed twice in Hanks’ balanced salt solution (HBSS), and incubated for 30 min at room temperature.

### DRG neuron culture and imaging

Primary cultures of DRG neurons were generated from C57BL/6N mice as described previously (*65*). Briefly, DRG was incubated in collagenase/dispase (5 mg/ml) (10269638001, Millipore-Sigma) for 30 min, and cells were mechanically dissociated, seeded on poly-d lysine–coated coverslips, and cultured for 48 hours [Dulbecco’s modified Eagle medium (DMEM)/F12, 10% fetal bovine serum (FBS), penicillin/streptomycin, nerve growth factor (100 ng/ml), and glial cell line–derived neurotrophic factor (50 ng/ml)]. For calcium imaging, DRG neurons were transfected with AAV9.Syn.GCaMP6f.WPRE.SV40 (2 μl, 1 × 10^13^ μg/ml, AV-9-PV2824) (*66*). After 48 hours of culture, coverslips with DRG neurons expressing the calcium indicator GCaMP6f were mounted on a DMi8 microscope (Leica) in HBSS buffer (140 mM NaCl, 2 mM CaCl_2_, 10 mM Hepes, 4 mM KCl, and 1 mM MgCl_2_ (pH 7.4)] and constantly superfused from a gravity-fed six-channel system (VC-6, Warner Instruments). Imaging was performed with an ORCA-Flash 4.0 C1440 digital complementary metal-oxide semiconductor camera (Hamamatsu, Bridgewater, NJ) at 1 Hz. Fluorescence intensity in hand-drawn regions of interest was extracted using HCImage (Hamamatsu) and plotted against time. The R package Pheatmap was used to generate a heatmap plot to visualize fluorescence signals in all the neurons.

### Electrophysiology

Whole-cell patch clamp was performed on Mrgrpa3-Cre-eGFP neurons as previously described (*67*). Briefly, DRG neurons collected from Mrgpra3-Cre-eGFP mice were cultured on coverslips for 48 to 72 hours. Whole-cell current clamp recordings were performed on dissociated DRG neurons expressing eGFP. Data were recorded with an Axon 700B amplifier and pCLAMP 11 software (Molecular Devices, Sunnyvale). Pipette electrodes were pulled with P-97 microelectrode puller (Sutter Instrument) from borosilicate glass (World Precision Instruments Inc.) with resistances of 2 to 4 megohms. The extracellular solution contained 140 mM NaCl, 4 mM KCl, 2 mM CaCl_2_, 1 mM MgCl_2_, 10 mM Hepes, and 10 mM glucose with a pH of 7.4 and an osmolality of 300 mosm/kg. The pipette solution contained 140 mM KCl, 1 mM MgCl_2_, 1 mM EGTA, 10 mM Hepes, 3 mM adenosine 5′-triphosphate, and 0.5 mM guanosine 5′-triphosphate with a pH of 7.4 and an osmolality of 300 mosm/kg.

### FLIPR screening

HEK293 cells were cultured in DMEM/F12 supplemented with 10% FBS, penicillin (100 U/ml), and streptomycin (100 μg/ml). For transient expression, 8 × 10^5^ cells were seeded, cultured for 24 hours, and transfected using TransIT-293 (Mirus Bio). Cells were transfected with GCaMP6s, Gα15 subunit, together with an individual MRG receptor. Expression vectors for MRGPRX1, MRGPRX2, MRGPRX3, MRGPRX4, MRGPRD, MRGPRE, MRGPRF, and MRGPRG were obtained from Addgene. Mouse Mrgpr expression vectors for Mrgpra1, Mrgpra2b, Mrgpra3, Mrgprb2, Mrgprb4, Mrgprc11, and Mrgprd were obtained from X. Dong at Johns Hopkins University (*68*) as well as for Mrgpre (GenScript Inc.). After 48 hours, cells were plated at 20,000 cells per well in 96-well plates with HBSS (with calcium and magnesium) and measurements were made. The EC_50_ values were determined with a three-parameter dose-response curve in GraphPad Prism.

### Peptide net charge value calculation

The net charge *Z* of a peptide at a certain pH can be estimated by calculating

$$z=\sum_{i}N_{i}\frac{10^{\mathrm{pKi}}}{10^{\mathrm{pH}}+10^{\mathrm{pKi}}}-\sum_{j}N_{j}\frac{10^{\mathrm{pH}}}{10^{\mathrm{pH}}+10^{\mathrm{pKj}}}$$

where *N_i_* are the number and pK*_i_* are the p*K*_a_ values of the N terminus and the side chains of arginine, lysine, and histidine. The *j*-index pertains to the C terminus and the aspartate, glutamate, cysteine, and tyrosine (*69*).

### In situ hybridization

In situ hybridization was performed using the RNAscope technology (Advanced Cell Diagnostics, Newark, CA) according to the manufacturer’s instructions as previously described. Mouse ears were embedded into cryostat embedding media, freshly frozen, and then cryosectioned at 20-μm thickness. Images were collected on an Eclipse Ti (Nikon, Melville, NY) confocal laser-scanning microscope.

### Statistical analysis

Prism 7.0 (GraphPad Software) was used for statistical analyses. Differences between mean values were analyzed using two-tailed Student’s *t* test, paired *t* test, or analysis of variance (ANOVA) with post hoc analysis suggested by Prism. Differences were considered significant for *P < 0.05. Exact *P* values, definition and number of replicates, as well as definitions of center and dispersion are given in the respective figure legend. No statistical method was used to predetermine sample sizes.
